# Deep learning in assisting dermatologists in classifying basal cell carcinoma from seborrheic keratosis

**DOI:** 10.3389/fonc.2025.1507322

**Published:** 2025-04-24

**Authors:** Li-Hong Mei, Meng-Ke Cao, Jing Li, Xuan-Guang Ye, Xiang-Dong Liu, Gao Yang

**Affiliations:** ^1^ Department of Dermatology, Jinshan Hospital of Fudan University, Shanghai, China; ^2^ Department of Dermatology, Shanghai Eighth People’s Hospital, Shanghai, China; ^3^ Department of Pathology, Jinshan Hospital, Fudan University, Shanghai, China; ^4^ Department of Dermatology, Shanghai Sixth People’s Hospital, Jiaotong University, Shanghai, China

**Keywords:** basal cell carcinoma, seborrheic keratosis, deep learning, dermatologist assistance, skin cancer diagnosis

## Abstract

**Objectives:**

This study aimed to evaluate the effectiveness of deep learning model in assisting dermatologists in classifying basal cell carcinoma (BCC) from seborrheic keratosis (SK). The goal was to assess whether AI-assisted diagnostics could improve accuracy, reduce misdiagnoses, and potentially enhance clinical outcomes.

**Methods:**

This prospective study included 707 patients with histopathologically confirmed BCC or SK as an internal dataset (validation cohort), along with 5572 patients from the ISIC public dataset as an external dataset (split into training and test cohort). The images were preprocessed and augmented before being fed into a deep learning model based on the CLIP ViT-B/16 architecture. The model’s performance was assessed using the area under the receiver operating characteristic (ROC) curves (AUC). Two dermatologists, one with 3 years of experience and another with 15 years of experience, reviewed the cases before and after receiving the deep learning model’s predictions. Net reclassification index (NRI) and integrated discrimination improvement (IDI), was used to quantify the improvement in reclassification performance.

**Results:**

The model achieved an AUC of 0.76 in the training cohort and 0.79 in the test cohort for differentiating between BCC and SK. In the validation cohort, the model demonstrated an AUC of 0.71. Dermatologist 1’s AUC improved from 0.75 to 0.82 with deep learning model assistance, while Dermatologist 2’s AUC increased from 0.79 to 0.82. NRI and IDI analysis revealed statistically significant improvements, with Dermatologist 1 showing a 18% improvement and Dermatologist 2 showing a 11% improvement. Additionally, attention mechanisms like Grad-CAM provided insights into the model’s decision-making process, enhancing the interpretability of its predictions.

**Conclusion:**

The deep learning model demonstrated significant potential in aiding dermatologists in classifying BCC from SK.

## 1 Introduction

Basal cell carcinoma (BCC) is the most prevalent form of skin cancer, accounting for approximately 75% of non-melanoma skin cancers globally. Early and accurate diagnosis of BCC is crucial to ensure appropriate treatment and to prevent progression (1). Seborrheic keratosis (SK) is a benign lesion (2). Differentiating SK from pigmented BCC can be clinically challenging, particularly in individuals with dark skin. Dermoscopically, pigmented basal cell carcinoma (BCC) in darker skin can manifest as pigmented nodules, patches, or plaques, as well as ulcers, red nodules, red patches or plaques, or lesions resembling scars, depending on the specific BCC subtype and location on the body (3).

Current diagnostic methods of BCC and SK, including dermoscopy and histopathological examination, which are reliable but time-consuming. Additionally, dermoscopic interpretation depends heavily on the expertise of the dermatologist, which introduces a risk of human error, particularly in ambiguous cases (4). As a result, the need for non-invasive, efficient, and accurate diagnostic tools is becoming a clinical urgent.

Deep learning is a subset of artificial intelligence, which has made significant strides in the field of medical diagnostics in image classification, particularly. Deep learning models can be trained to identify patterns in medical images, enabling them to assist healthcare professionals in diagnosis. In dermatology, deep learning models have demonstrated remarkable accuracy in distinguishing between various types of skin lesions, offering a promising tool for reducing diagnostic workload and improving accuracy (5). Deep learning models are particularly well-suited to tackle image classification tasks, which is critical in the potential useful in differentiating benign lesions from malignant ones (6, 7). However, despite these advancements, there is a gap in the literature regarding the application of deep learning to the specific problem of assisting dermatologists in classifying BCC from SK. These two conditions that can appear visually similar (3, 8).

We assumed that deep learning can improve diagnostic accuracy to achieve this task. Thus, the aim of this study is to evaluate the performance of deep learning algorithms in assisting dermatologists in classifying BCC from SK. By comparing the diagnostic accuracy of dermatologists with and without the assistance of deep learning model, we seek to determine whether deep learning can serve as a valuable decision-support tool. The successful integration of these models into clinical practice could potentially reduce unnecessary biopsies and enhance early detection of BCC, improving overall patient care.

## 2 Materials and methods

### 2.1 Ethics

This prospective study adhered to the principles outlined in the Declaration of Helsinki and received approval from the Institutional Review Board of Jinshan Hospital (JIEC 2023-S85). Prior to enrollment, all participants provided written informed consent for the publication of any potentially identifiable data or images.

### 2.2 Dataset

Between November 1, 2023, and November 1, 2024, adult patients who underwent surgical resection for skin neoplasm were included in the study. The inclusion criteria were: (1) Histopathologically confirmed BCC or SK; and (2) Age ≥ 18 years. Exclusion criteria were: (1) Presence of systemic infection; (2) Incomplete clinical data; and (3) Images with motion blur or artifacts. A total of 707 patients with BCC or SK were ultimately enrolled, forming the internal dataset. The image were captured by both dermoscopy and smart devices with cameras. Additionally, the international skin imaging collaboration dataset (ISIC, https://www.isic-archive.com) was accessed with the same inclusion criteria. Images with visible artifacts or interference from nearby structures were excluded. Finally, 5,572 patients with BCC or SK in the external dataset (images from both dermoscopy and other devices were included).

### 2.3 Data split

The external ISIC dataset was divided into training and test cohorts in a 9:1 ratio. The internal hospital dataset was used as a validation cohort to evaluate the model’s performance and verify the effectiveness of deep learning model in assisting dermatologists in classifying BCC from SK.

### 2.4 Preprocessing and image resizing

All images were resized to a standard size compatible with the input layer of the deep learning model and converted into tensor format. Additionally, the preprocessing phase involved data augmentation techniques such as random cropping, rotation, flipping, and color adjustments. Random cropping provided image information from varying locations, while rotation and flipping helped the model recognize objects from different orientations. Meanwhile, color adjustments increased the model’s robustness to changes in lighting and color variations.

### 2.5 Normalization

The normalization process involved subtracting the mean value of the entire dataset and dividing by the standard deviation, bringing pixel values into a standardized range. This operation minimized brightness differences and color biases between images, allowing the model to focus on relevant features rather than noise or irrelevant details. By normalizing the data, the issues of gradient explosion and vanishing gradients during training were effectively mitigated, leading to improved model stability and faster convergence. Additionally, normalization ensured that the model remained sensitive to the scale of input data, enhancing its robustness when handling images of varying scales.

### 2.6 Deep learning model architecture

The deep learning model utilized was based on the multimodal pre-trained CLIP model (9), employing the ViT-B/16 architecture (10). In the ViT-B/16 architecture, images were divided into fixed-size patches, with each patch flattened and embedded into a vector. These vectors were then fed into the Transformer encoder to capture global image relationships. Using the self-attention mechanism, the Transformer learns connection between different image regions and generates a comprehensive image representation. This representation was subsequently processed through several fully connected layers to produce the final classification or regression outputs.

### 2.7 Feature extraction

The feature extraction process of the CLIP model converted raw image data into normalized high-dimensional feature vectors. The input images were divided into fixed-size patches, and each patch was mapped to a low-dimensional space via a learnable linear projection, creating an embedding vector for each patch. To capture the spatial relationships between the patches, position encodings were added to these embedding vectors. The Transformer encoder was then employed to process the sequence of embedding vectors, utilizing self-attention mechanisms and fully connected feed-forward networks to extract semantic information and contextual relationships between image patches. After encoding, a fixed-length vector representation was obtained through feature pooling, which was then projected via a fully connected layer into the same embedding space as the text features. In addition, CLIP simultaneously learned semantic information from both images and text.

### 2.8 Training

The model was trained using the stochastic gradient descent (SGD) optimizer, a widely-used method for deep learning tasks that works by updating the model’s weights at each iteration to minimize the loss function. To enhance convergence speed, SGD with momentum was applied, helping the model reach local optima more efficiently during training. The initial learning rate was set at 0.002, with a momentum value of 0.9 and a weight decay parameter of 0.005.

### 2.9 Loss function

The loss function used was CrossEntropy Loss, which quantified the difference between the model’s predictions and the true labels. In this function, the model’s outputs were first passed through a softmax layer to convert them into a probability distribution. These predicted probabilities were then compared to the true labels to calculate the cross-entropy loss, which measured the negative log-likelihood of the correct class. For each sample, the loss reflected the confidence of the model in its prediction for the true label. The goal of this loss function was to minimize the disparity between the predicted probability distribution and the true labels, thus enabling the model to improve its accuracy in classifying the samples.

### 2.10 Training process

The training approach employed CoOp, a parameter-efficient fine-tuning technique (11). CoOp focused on freezing the parameters of the model backbone while introducing a learnable text prompt parameter at the input of the text encoder. This parameter was learned during training, allowing it to adapt to specific downstream tasks, thus achieving effective fine-tuning with minimal computational resources. Following the prompt-learning methodology of CLIP, each class name was integrated into a prompt, generating a set of prompts for the text encoder in a given classification task. Each prompt included a common set of learnable parameters along with a specific class name. These prompts were fed into the text encoder to generate corresponding feature vectors. Simultaneously, training images were processed through the image encoder to produce their respective feature vectors. The next step involved calculating the normalized similarity between the image feature vector and each output feature vector from the text encoder. Finally, CrossEntropy Loss was computed against the ground truth labels to optimize the model’s performance. A linear classifier was attached to the CLIP image encoder as an evaluation module for the downstream task.

### 2.11 Evaluation metrics

To explore the clinical application of the deep learning model, two dermatologists (dermatologist 1 with 3 years of experience and dermatologist 2 with 15 years of experience) reviewed the images of each case to identify BCC or SK without access to histopathological data. Afterward, the dermatologists were presented with the classification results generated by the deep learning model. Any reclassification decisions made by the dermatologists following the presentation of the model’s results were documented. The area under the receiver operating characteristic (ROC) curves (AUC) were used to evaluate the clinical application of the deep learning model in assisting dermatologists. Attention mechanisms were employed to visualize the regions of interest that the model focused on during image recognition. Specifically, attention maps were generated to illustrate which parts of the images the model emphasized, such as color, shape, or texture. Gradient-weighted Class Activation Mapping (Grad-CAM) was used to highlight the areas in the images that the model deemed most important for predicting the corresponding labels. Additionally, an analysis was conducted on cases where the model underperformed or failed. This included investigating the characteristics of such cases and identifying potential reasons for the model’s performance issues.

### 2.12 Computational requirements and runtime

Our server was equipped with two NVIDIA RTX 4090 GPUs, each offering 24 GB of memory. The central processing unit (CPU) was an Intel Core i9-13900K with 24 cores and 36 threads. The system had 256 GB of DDR4 RAM, ensuring ample memory for large datasets and computational tasks. Data storage was handled by a 1 TB SSD, which provided high-speed data read and write operations. The operating system was Ubuntu 20.04, and the software environment included key tools and libraries such as PyTorch 1.10, CUDA 11.2, Anaconda 23.3.1, CUDA 12.0, cuDNN v8.8.1, PyTorch 1.13.1, and Python 3.7.16. For the public ISIC dataset, the total training time for 100 epochs was around 1.1 hours, and the average inference time per instance was 20.50 milliseconds. For the internal dataset, the total runtime for 100 epochs was approximately 3 minutes, and the average inference time per instance was 2.56 milliseconds.

### 2.13 Clinical usefulness analysis

The net reclassification index (NRI) and the integrated discrimination index (IDI) were calculated to compare the discrimination performance between the dermatologists’ classifications and the results assisted by the deep learning model. These metrics provided insight into how well the model improved diagnostic accuracy and reclassification compared to human performance alone.

### 2.14 Statistical analysis

Statistical analysis was conducted using R software (version 4.3.2; https://www.r-project.org/). The normality of the data was assessed using the Shapiro-Wilk test, and the homogeneity of variance was evaluated using Levene’s test. For continuous variables, independent-samples t-tests were used to compare groups if the data met the assumptions of normality and homoscedasticity. In cases where these assumptions were not satisfied, the non-parametric Mann-Whitney U test was applied. Categorical variables were analyzed using the chi-squared test or Fisher’s exact test, depending on the expected cell counts. A p-value of less than 0.05 was considered statistically significant in all analyses.

## 3 Results

### 3.1 Data sets

**Figure 1:**
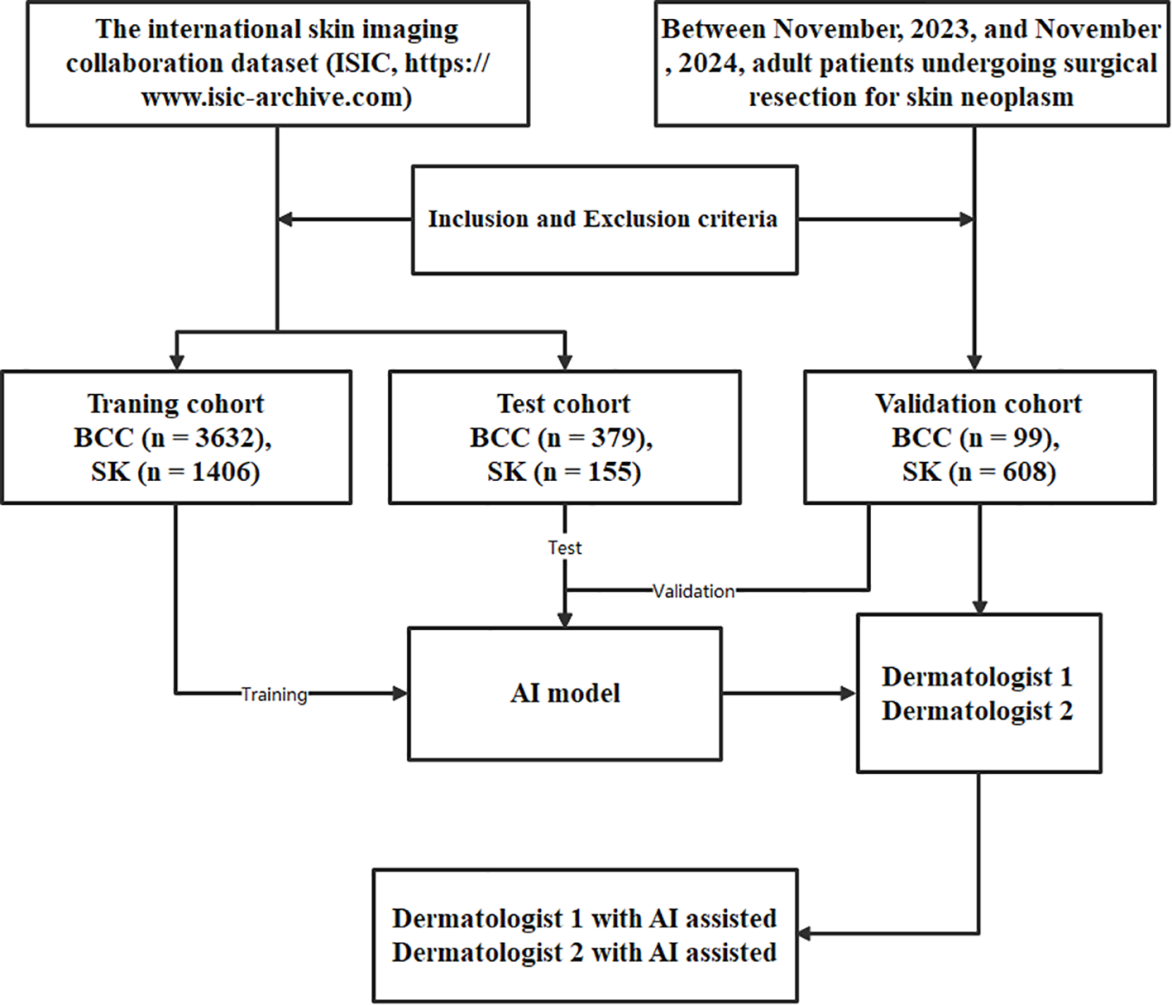
The process flow for the deep learning model developed to classify basal cell carcinoma (BCC) and seborrheic keratosis (SK). This flow includes data collection, preprocessing, model training, feature extraction, classification, reclassification, and validation.

**Figure 2:**
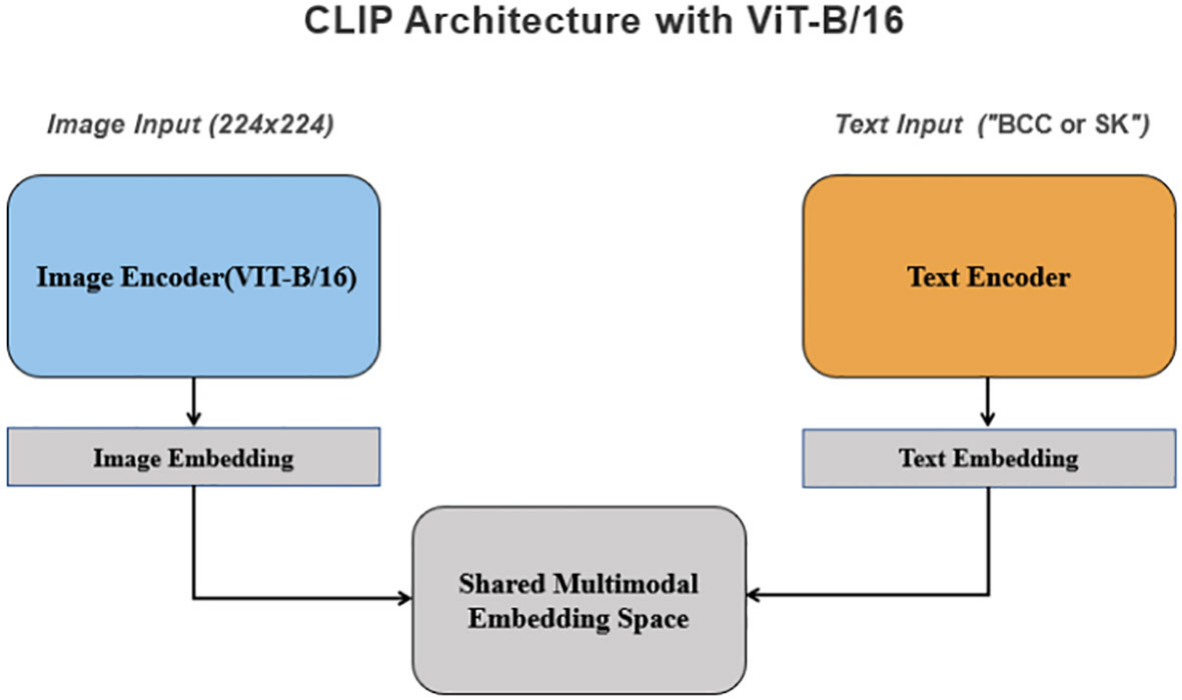
The structure and process flow of the deep learning model used for distinguishing BCC and SK, based on the CLIP architecture with ViT-B/16. Images are divided into uniform patches, embedded, and processed through a Transformer encoder that captures relationships across image regions to produce a comprehensive representation.

The workflow of this study is illustrated in **Figure 1**. Two case examples of BCC and SK are provided in **Figure 2**. The training dataset (external dataset) consisted of 5038 patients (1916 females and 3113 males with 9 missing), with an average age of 65 ± 14.6 years (range 20-85). This included 554 females and 848 males in the SK group (aged 63 ± 14.3 years, range 20-85) and 1362 females and 2265 males (5 missing) in the BCC group (aged 66 ± 14.6 years, range 20-85).

The test dataset (external dataset) contained 534 patients (200 females and 334 males), with an average age of 65 ± 15.6 years (range 20-85). Within the SK group, there were 135 females and 244 males (aged 66 ± 15.3 years, range 25-85), while the BCC group had 65 females and 90 males (aged 62 ± 15.9 years, range 25-85).

The validation cohort (internal dataset) included a total of 707 patients (389 females and 318 males), with an average age of 56 ± 13.6 years (range 21-91). Among these patients, 610 were diagnosed with SK and 97 with BCC. Specifically, the SK group included 328 females and 280 males (aged 56 ± 13.4 years, range 21-91), while the BCC group consisted of 61 females and 38 males (aged 60 ± 14.2 years, range 25-88).

### 3.2 Model performance in the training and test cohort

**Figure 3:**
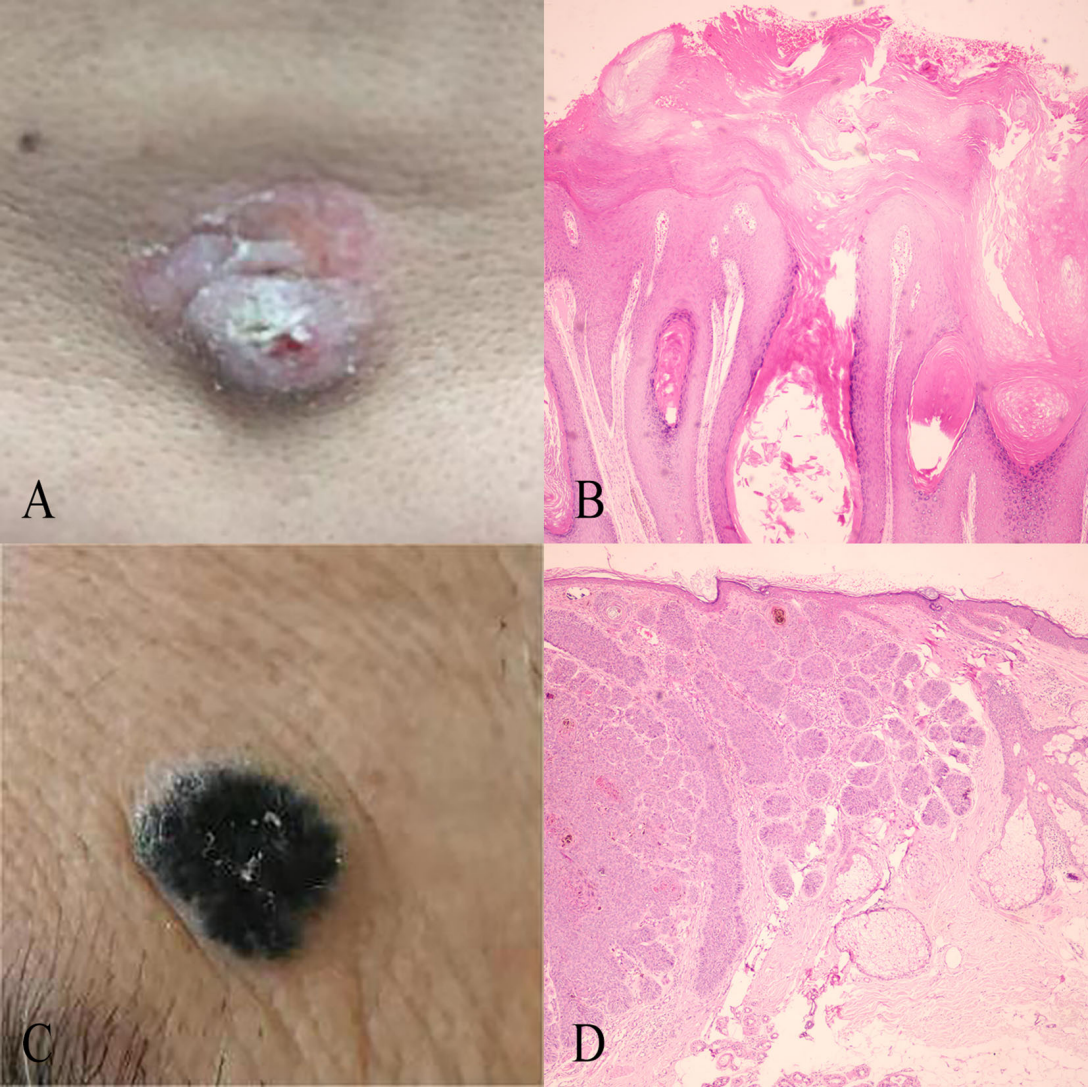
Case examples of BCC and SK classification. **(A)** An incorrectly classified BCC lesion by Dermatologist 2, which was correctly reclassified as BCC with the model’s assistance, matching the histopathological diagnosis. **(B)** An incorrectly classified SK lesion by Dermatologist 2, correctly reclassified as SK using the model, with alignment to histopathological findings. **(C)** Histopathological findings of the BCC case in **(A)**, showing typical features such as atypical keratinocytes, parakeratosis, and an irregular stratum corneum. **(D)** Histopathological findings of the SK case in **(B)**, displaying features like acanthosis, hyperkeratosis, and horn cysts.

The architecture of the deep learning model is depicted in **Figure 3**. The initial performance metrics in the training cohort revealed an AUC of 0.76, reflecting a moderate discrimination ability to differentiate between BCC and SK. The model showed a sensitivity of 0.58 and a specificity of 0.94, with positive predictive value (PPV) and negative predictive value (NPV) of 0.79 and 0.85, respectively. In the test cohort, the model exhibited an AUC of 0.79. The model achieved a sensitivity of 0.65 and a specificity of 0.94, with a PPV of 0.81 and an NPV of 0.87. For the validation cohort, the model exhibited an AUC of 0.71, with a sensitivity of 0.92,a specificity of 0.51, a PPV of 0.92 and a NPV of 0.52 (**Table 1**).

**Table 1:** Area under the curve (AUC) for model performance and dermatologist assessments.

		AUC	95%CI	SPE	SEN	NPV	PPV
Training cohort	DL model	0.76	0.75-0.77	0.94	0.58	0.85	0.79
Test cohort	DL model	0.79	0.75-0.83	0.94	0.65	0.87	0.81
Validation cohort	DL model	0.71	0.66-0.76	0.51	0.92	0.52	0.92
	Dermatologist 1	0.75	0.71-0.8	0.66	0.85	0.42	0.94
	Dermatologist 1 with DL model	0.82	0.78-0.87	0.72	0.93	0.61	0.95
	Dermatologist 2	0.79	0.74-0.83	0.66	0.91	0.56	0.94
	Dermatologist 2 with DL model	0.82	0.77-0.86	0.69	0.95	0.67	0.95

AUC, Area Under the Curve; DL, deep learning; PPV, Positive Predictive Value; NPV, Negative Predictive Value; SEN, Sensitivity; SPE, Specificity.

### 3.3 The interpretability of the model

The results revealed that the model primarily relied on color features to classify sample categories. When making correct predictions, the model often focused on both color and shape characteristics of the target objects. Specifically, the model identified that SK and BCC shared a reliance on the background color (base) of the images. SK typically presented with light brown or brown hues, while BCC was characterized by light red or dark red shades. Additionally, surface features such as papules or patches were also key factors in the model’s classification. SK generally exhibited black papillomatous hyperplasia on the surface, whereas BCC often showed light-colored, damaged nodular hyperplasia. The smoothness of the skin surface also played a role in the model’s predictions, with SK typically having a smoother surface compared to the damaged and rough surface seen in BCC (**Supplementary Figure 1**).

The model’s misclassifications were attributed to four primary factors: Color blending: The color of the affected area was altered by surrounding skin or external influences, such as post-scratch erythema or sun exposure, causing a blend of colors. Superficial red coloration due to sunlight exposure might coexist with the brown base of seborrheic dermatitis, leading the model to mistakenly identify the erythema as basal erythema or capillary dilation. Blurry images: Image quality issues, such as blurriness from improper handling or equipment malfunction, affected the model’s ability to accurately recognize color and shape, which impacted prediction accuracy. Ensuring high-quality images is therefore crucial for improving the model’s performance. Interference from similar colors: The model sometimes struggled to differentiate between lesions that shared similar color features, such as BCC and SK. The close resemblance in color could confuse the model, resulting in mispredictions. Enhancing the model’s ability to distinguish these subtle differences will be essential in future training iterations. Small sample-specific features: Certain cases with unique characteristics, such as seborrheic skin with enlarged pores, posed challenges to the model’s predictions. Differences in skin smoothness between affected and normal skin could aid the model in making correct predictions, but when these differences were less pronounced, the model struggled (**Supplementary Figure 2**).

### 3.4 Comparative analysis with dermatologists and reclassification results

The internal dataset was used for comparative analysis with dermatologists and reclassification results in classifying SK from BCC. Dermatologist 1 achieved an AUC of 0.75 with sensitivity, specificity, PPV and NPV of 0.85, 0.66, 0.94, 0.42, respectively. Dermatologist 2 achieved an AUC of 0.79 with sensitivity, specificity, PPV and NPV of 0.91, 0.66, 0.94, 0.56, respectively. After incorporating the model’s prediction results, Dermatologist 1 achieved an AUC of 0.82 with sensitivity, specificity, PPV and NPV of 0.93, 0.72, 0.95, 0.61, respectively. Dermatologist 2 also showed an improved performance, with an AUC of 0.82 with sensitivity, specificity, PPV and NPV of 0.95, 0.69, 0.95, 0.67, respectively.

**Figure 4:**
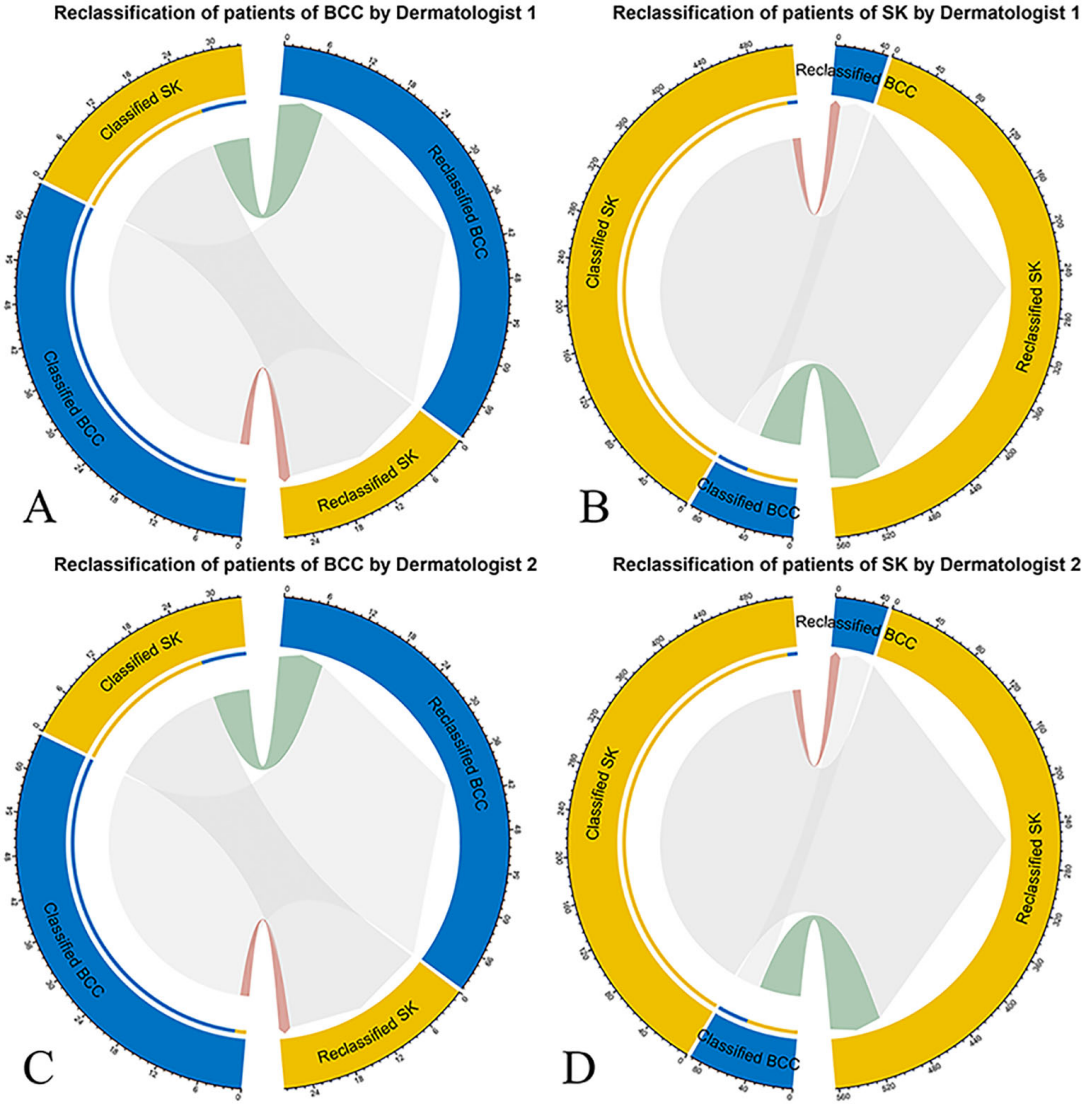
Net reclassification improvement (NRI) analysis for the deep learning model’s classification of BCC and SK. NRI measures the improvement in diagnostic accuracy with the model’s input compared to the initial assessments by dermatologists. Red connections represent patients incorrectly reclassified, while green connections indicate correct reclassifications, with patient numbers specified. **(A)** NRI for Dermatologist 1’s classification (left half circle) and reclassification (right half circle) of BCC with model assistance. **(B)** NRI for Dermatologist 1’s classification and reclassification of SK with model assistancel. **(C)** NRI for Dermatologist 2’s classification and reclassification of BCC. **(D)** NRI for Dermatologist 2’s classification and reclassification of SK with model assistance.

**Figure 5:**
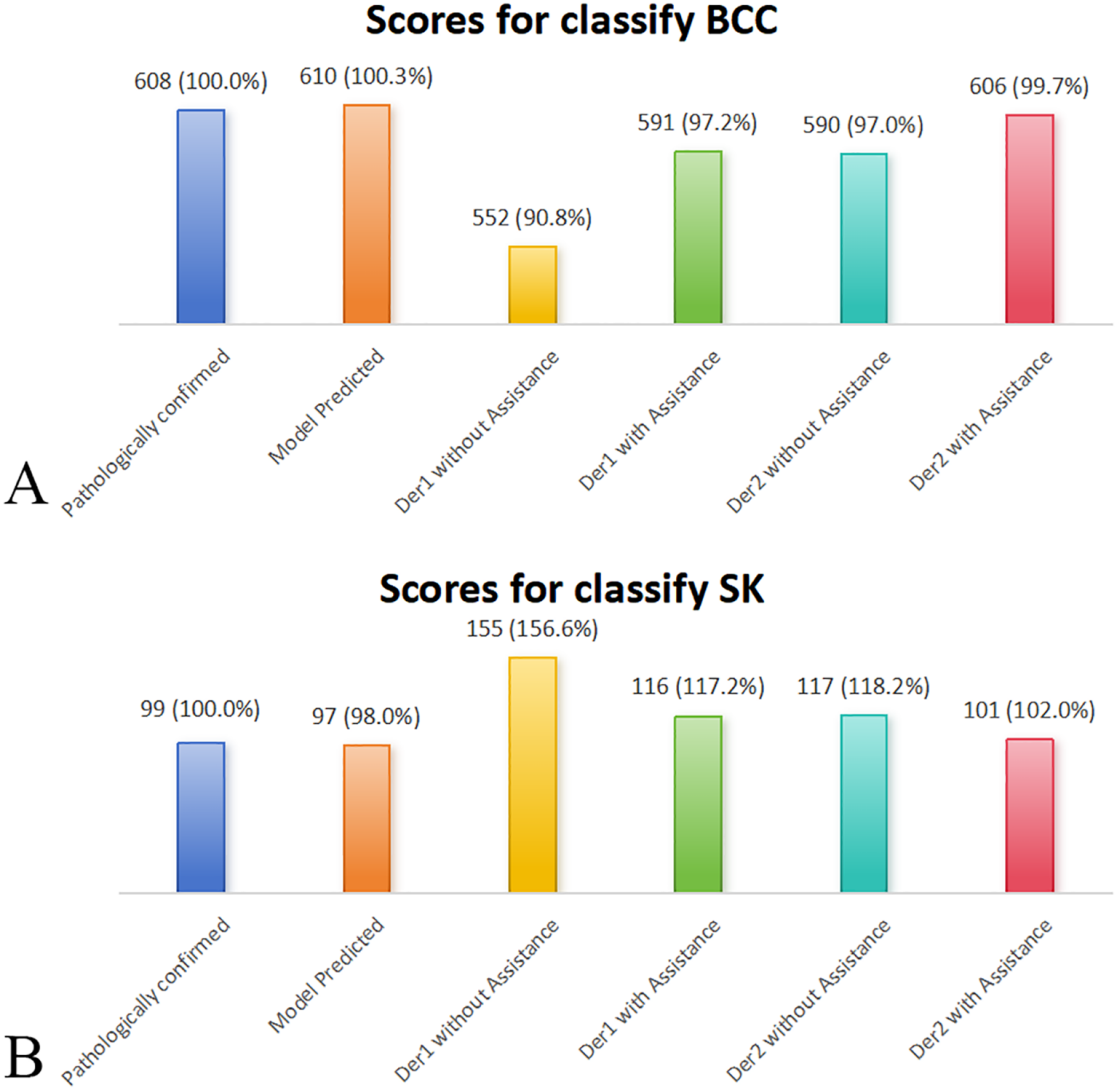
Comparative performance of the deep learning model, dermatologists (with/without deep learning model assistance), and pathologically confirmed cases (reference = 100%) on the external validation dataset. Bar plots display the classification scores for **(A)** BCC and **(B)** SK. Deep learning model assistance improved diagnostic accuracy for both dermatologists, bringing their scores closer to model and confirmed values. Der, dermatologist.

The NRI and IDI were 0.64 (P = 0.006) and 0.18 (P < 0.001) for Dermatologist 1 with the assistance of the model’s prediction results in discrimination between BCC and SK. The NRI and IDI were 0.06 (P = 0.049) and 0.11 (P < 0.001) for Dermatologist 2 with the assistance of the model’s prediction results in discrimination between BCC and SK (**Figure 4**). The score achieved by the model and dermatologists is shown in **Figure 5**.

## 4 Discussion

This study built and validated the capability of the multimodal pre-trained model CLIP in distinguishing between BCC and SK, achieving high accuracy across both the training, test and validation cohorts. The model’s robust performance is reflected in the AUC values, particularly in the test and validation cohorts, underscoring its high discriminative power. Furthermore, the deep learning model demonstrated significant potential in aiding dermatologists in classifying BCC from SK, improving diagnostic performance.

The result of this study is consistent with previous studies that have shown that transformer-based architectures, such as Vision Transformers (ViT), have demonstrated strong potential in medical image classification tasks, including dermatology (12). These models offer improved global context awareness over traditional CNNs, which is particularly beneficial in recognizing complex visual patterns present in pigmented lesions. Recent studies also demonstrated that transformer based models, when trained on large and diverse datasets, achieve high accuracy in classifying skin cancers (13). Transformers can outperform CNNs in identifying melanoma, BCC, and other pigmented skin disorders due to their ability to model long-range dependencies and attend to subtle lesion features (14). Our findings corroborate these studies by showing the deep learning model’s capacity to handle the task of distinguishing between BCC and SK with comparably to or exceeded the diagnostic accuracy of experienced dermatologists.

The reclassification analysis provided further evidence of the model’s utility. Both the NRI and IDI showed significant improvements, indicating that the model could effectively refine its predictions with additional data or through iterative adjustments (15). Dermatologist 1 and Dermatologist 2, after being assisted by the model, achieved AUCs of 0.80 and 0.89, respectively, indicating that deep learning models integration can enhance diagnostic capabilities and reduce the subjectivity inherent in human diagnosis. Interestingly, without the help of the deep learning model, dermatologists tended to diagnose ambiguous cases as benign SK. However, after seeing the prompts of the deep learning model, dermatologists tended to revise the previously uncertain cases into malignant BCC. This phenomenon was more significant in the junior dermatologist.

The study also underscores the importance of generalization in deep learning models, which is a critical factor for their clinical application. The ability of our model to maintain high performance across both the training and test cohorts reflects its adaptability. This is further reinforced by its performance in external validation datasets, which confirmed the model’s robustness when applied to new data. This generalization ability is vital for clinical applications, especially when preformed on diverse patient populations and image qualities (16). The consistent performance of the model across different cohorts suggests that the integration of deep learning into dermatological practice could yield significant clinical benefits. Deep learning models, including ours, typically exhibit high sensitivity and specificity, reducing the potential for misdiagnosis (17, 18).

Additionally, the attention-based mechanisms embedded in the CLIP model provided interpretable results, allowing us to visualize the areas of the images that the model focused on during classification. This feature is not only useful for improving the model’s transparency but also assists dermatologists in validating the deep learning models’ decisions. Similar techniques have been employed in other deep learning models to enhance diagnostic accuracy and interpretability in clinical settings. As a previous study incorporated deep learning models in clinical practice to further improve diagnostic performance (7).

One major limitation is the skewed gender distribution within the dataset, which may limit the generalizability of our findings across more diverse populations. Expanding the dataset to include a broader demographic and geographic range will be important for enhancing the model’s robustness. In addition, variations in image acquisition protocols, device resolutions, and population demographics across institutions may influence the performance of deep learning models. Future studies should incorporate with detailed information on imaging devices to better understand and mitigate these effects. Moreover, integrating multi-modal data, such as patients’ clinical histories or histopathological information, could further improve the model’s diagnostic performance (9). Furthermore, to guarantee the model’s practical utility and its tangible effects in real-world scenarios, it is crucial to validate it across a range of clinical environments that have different levels of institutional resources.

## 5 Conclusion

In conclusion, this study demonstrates that the CLIP-based deep learning model can significantly enhance the diagnostic capabilities of dermatologists when distinguishing between BCC and SK.

## Data Availability

The raw data supporting the conclusions of this article will be made available by the authors, without undue reservation.

## Supplementary material

Supplementary Figure 1 The results revealed that the model primarily relied on color features to classify sample categories. When making correct predictions, the model often focused on both color and shape characteristics of the target objects. Specifically, the model identified that SK and BCC shared a reliance on the background color (base) of the images. SK typically presented with light brown or brown hues, while BCC was characterized by light red or dark red shades. Additionally, surface features such as papules or patches were also key factors in the model’s classification. SK generally exhibited black papillomatous hyperplasia on the surface, whereas BCC often showed light-colored, damaged nodular hyperplasia. The smoothness of the skin surface also played a role in the model’s predictions, with SK typically having a smoother surface compared to the damaged and rough surface seen in BCC.

Supplementary Figure 2 These insights underscore the importance of addressing factors such as color blending, image quality, and feature similarity in future model training to improve its prediction accuracy and robustness.
