# Supplementary figures and images for: Deep learning in assisting dermatologists in classifying basal cell carcinoma from seborrheic keratosis
Source: Front Oncol. 2025 Apr 24;15:1507322. doi: 10.3389/fonc.2025.1507322 (PMC12058839; doi:10.3389/fonc.2025.1507322)

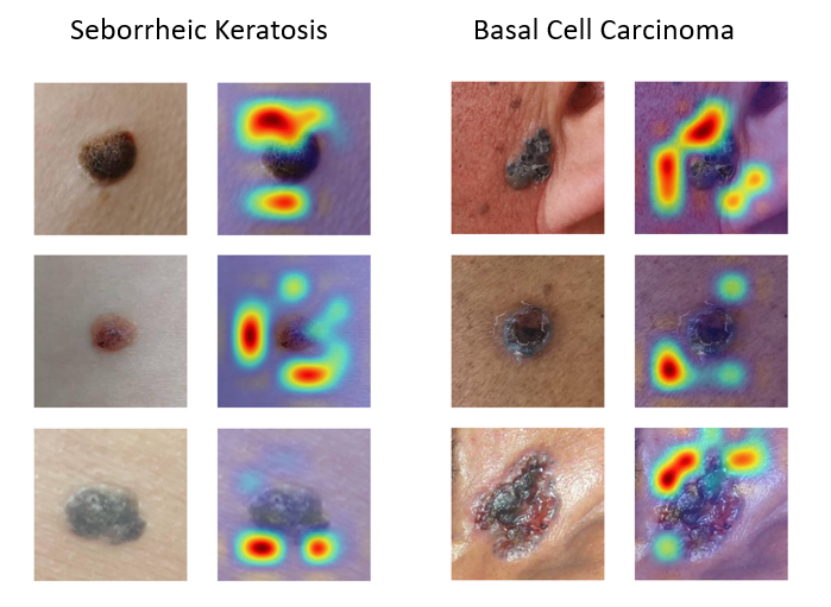

Supplement: Supplementary Figure 1 — The results revealed that the model primarily relied on color features to classify sample categories. When making correct predictions, the model often focused on both color and shape characteristics of the target objects. Specifically, the model identified that SK and BCC shared a reliance on the background color (base) of the images. SK typically presented with light brown or brown hues, while BCC was characterized by light red or dark red shades. Additionally, surface features such as papules or patches were also key factors in the model’s classification. SK generally exhibited black papillomatous hyperplasia on the surface, whereas BCC often showed light-colored, damaged nodular hyperplasia. The smoothness of the skin surface also played a role in the model’s predictions, with SK typically having a smoother surface compared to the damaged and rough surface seen in BCC. [file Image1.tiff]

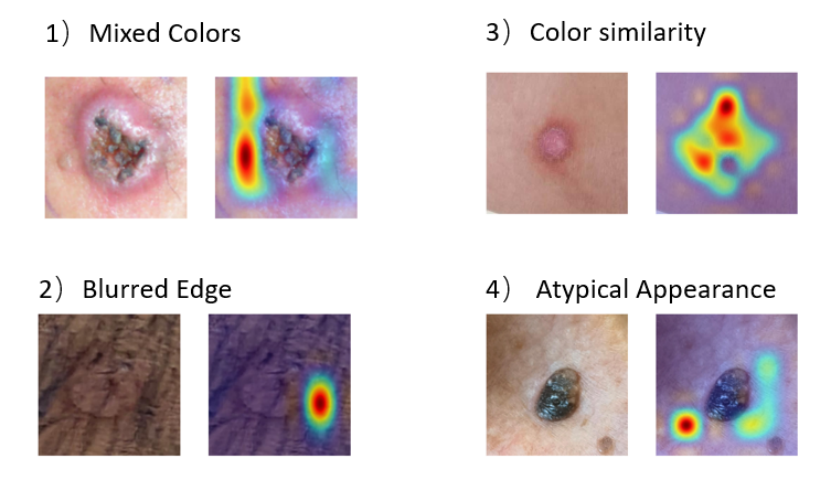

Supplement: Supplementary Figure 2 — These insights underscore the importance of addressing factors such as color blending, image quality, and feature similarity in future model training to improve its prediction accuracy and robustness. [file Image2.tiff]
